# Tumor penetrating nanomedicine targeting both an oncomiR and an oncogene in pancreatic cancer

**DOI:** 10.18632/oncotarget.27160

**Published:** 2019-09-03

**Authors:** Maud-Emmanuelle Gilles, Liangliang Hao, Kaelyn Brown, Jihoon Lim, Sangeeta N. Bhatia, Frank J. Slack

**Affiliations:** ^1^ HMS Initiative for RNA Medicine, Department of Pathology, Cancer Center, Beth Israel Deaconess Medical Center, Harvard Medical School, Boston, Massachusetts, USA; ^2^ Institute for Medical Engineering and Science, Massachusetts Institute of Technology, Cambridge, Massachusetts, USA; ^3^ Koch Institute for Integrative Cancer Research, Massachusetts Institute of Technology, Cambridge, Massachusetts, USA; ^4^ Department of Medicine, Brigham and Women’s Hospital and Harvard Medical School, Boston, Massachusetts, USA; ^5^ Broad Institute of Massachusetts Institute of Technology and Harvard, Cambridge, Massachusetts, USA; ^6^ Howard Hughes Medical Institute, Cambridge, Massachusetts, USA; ^7^ Marble Center for Cancer Nanomedicine, Massachusetts Institute of Technology, Cambridge, Massachusetts, USA

**Keywords:** miRNAs, KRAS, RNA-based-therapy, PDAC, tumor-penetrating nanocomplexes

## Abstract

Developing new targeted therapy for pancreatic cancer is one of the major current challenges in cancer research. KRAS mutations and miRNA dysregulation (e.g. miR-21-5p oncomiR) play key roles in Pancreatic Ductal Adenocarcinoma (PDAC), leading to rapid progression of the disease. As the KRAS mutation is a main driver of PDAC, anti-KRAS strategies remain a major therapeutic approach for PDAC treatment. Previously, utilization of either siKRAS or small chemically modified single-stranded RNA molecules that specifically disable miR-21 (anti-miR-21) were effective in slowing PDAC tumor growth in various tumor models when packaged in an innovative delivery system (TPN) required for efficient drug delivery to the PDAC tumor site. Here we have tested the utility of targeting the KRAS pathway through multiple mechanisms and via dual targeting of a PDAC oncomiR and oncogene. Initially we found that miR-217, which has been shown to directly regulate KRAS expression, is downregulated in our PDAC samples, thus we tested the benefits of anti-miR-21, miR-217 mimic or siKRAS loaded into the tumor-penetrating nanoparticles (TPN) that we had previously shown to potently target the largely impenetrable PDAC tumors, and found an enhanced anti-tumoral response upon dual treatments in KRAS-mutated PDAC models.

## INTRODUCTION

Pancreatic cancer is currently the second leading cause of all cancer-related deaths, with a dramatically low 5-year survival associated with a poor prognosis [1, 2]. In addition to improvement of early detection, the development of potent targeted therapy methods is desperately needed. In the past, utilization of single-agent treatments like gemcitabine or combination treatments like FOLFORINOX (5-fluorouracil (5-FU), irinotecan, oxaliplatin, and leucovorin) has allowed non-resistant patient response and increased survival for metastatic patients [1]. Combinations of multiples drugs have emerged as possible therapies and the combination of Gemcitabine with erlotinib or with nab-paclitaxel has been beneficial for patients [3, 4]. Multiple other approaches are currently being developed for PDAC targeted therapies including the targeting of key regulators of the disease like oncogenes or small non-coding RNA (e.g. miRNAs).

PDAC tumors are characterized by a strong desmoplastic reaction (rich stroma) that impairs easy drug delivery into the tumor site, leading to insufficient tumor targeting. Recently, various strategies have been developed to improve drug delivery to PDAC sites by utilizing tools such as nanoparticle albumin-bound paclitaxel, pseudopeptides, or polyplexes [3, 5, 6]. Additionally, various engineering approaches have led to the safe delivery of therapeutic compounds into tumor sites; among them, iRGD-guided tumor penetrating nanocomplexes (TPNs) have been specifically successful [7, 8]. TPNs promote the safe delivery of RNA-based therapeutics including single-strand oligonucleotides [9, 10]. We have successfully used TPNs for delivery to PDAC of an RNA-based therapeutic (antimiR-21) that targets the oncomiR (miR-21-5p) named TPN-21. TPN-21 limits cell growth and increases apoptosis in various PDAC models including patient derived organoids (PDO) and patient derived xenografts (PDX), allowing its use for personalized medicine [9]. Along the same lines, due to the prevalence of *KRAS* mutations in PDAC, the *KRAS* oncogene has been targeted using an siRNA (si-KRAS) by loading it in similar particle formulations [10, 11]. Utilization of iRGD TPNs carrying *KRAS* siRNA also led to the limiting of tumor growth *in vivo* [12]*.* Because miR-21 overexpression and *KRAS* mutation have been extensively reported to be a part of a PDAC signature with a strong clinical correlation for PDAC progression and survival [9, 10, 13], we hypothesized that dual targeting of these two key players could improve the anti-cancer effects.

Since it seems that *KRAS* knockdown is unlikely to suffice as a monotherapy given the strong possibility of resistance due to compensatory mutations and altered expression profiles, it is necessary to identify and test new targets that will enhance or synergize with *KRAS* pathway blockades. Gene expression screens, shRNA screens and CRISPR/Cas9-powered screens are generating unprecedented lists of genetic targets that have the potential to become new RNA therapies [14].

We and other have found miR-217-5p to be downregulated in PDAC samples and its dysregulation has been found to be significantly associated with low survival in a variety of cancer types (Table 1) [9, 15].

**Table 1:** Expression of natural KRAS targeting miRNA (miR-217-5p) is associated with poor overall survival in cancers and could be reintroduced or targeted for therapy

Cancer Type	*P* Value	miR-217 expression associated low survival
Kidney renal papillary cell carcinoma [KIRP]	8.79E-05	high expression
Bladder Urothelial Carcinoma [BLCA]	0.00033535	high expression
Colon adenocarcinoma [COAD]	0.00609379	high expression
Kidney Chromophobe [KICH]	0.03016767	high expression
Brain Lower Grade Glioma [LGG]	4.70E-10	high expression
Mesothelioma [MESO]	0.00293455	high expression
Stomach adenocarcinoma [STAD]	0.02206944	high expression
Ovarian serous cystadenocarcinoma [OV]	0.06356434	low expression
Testicular Germ Cell Tumors [TGCT]	0.04835562	low expression

Table 1 has been generated from the PROGmiR tool by querying miR-217-5p expression level and correlating this with overall survival (p-value £ 0.1) among 33 human cancer databases. Non-significant results (p-value ³ 0.1) are not presented in the table.

miR-217 is also known to bind to the *KRAS* 3′UTR and impair its expression, leading to tumor suppressor activities in various cancers like acute myeloid leukemia, colorectal cancer and PDAC [16–18]. Because *KRAS* remains a notoriously undruggable target, here we explore various approaches such as the use of miRNA targeting *KRAS* (miR-217 mimic) for successful combination therapy with antimir-21. As TPN approach has been successful to deliver antimiR in PDAC model, subject to validation of the anti-tumor effect of miR-217 mimic *in vitro*, *In vivo* reintroduction of the mimic will be performed through used of TPN.

In this study, we tested dual targeting of miR-21 (anti-miR-21) and *KRAS* (siKRAS or mimic-217) packaged in TPNs for PDAC therapy. We first highlight the *KRAS* and miRNAs signature of our PDAC mouse model as well as the influence of *KRAS* knock-down on miR-21 expression. We then evaluated the best approaches to target *KRAS* in our model by using chemically modified double-stranded RNAs that mimic endogenous miRNA miR-217 known to bind the *KRAS* 3′UTR and impair its expression. Then we evaluated the combination of an siRNA against *KRAS* and anti-mir-21 loaded into the TPN for gymnotic delivery *in vitro* and in an organoid model *in vitro,* as well as for systemic intravenous injection *in vivo*.

## RESULTS

### *KRAS* mutation and miRNA dysregulation are components of the mPDAC gene signature

**Figure 1:**
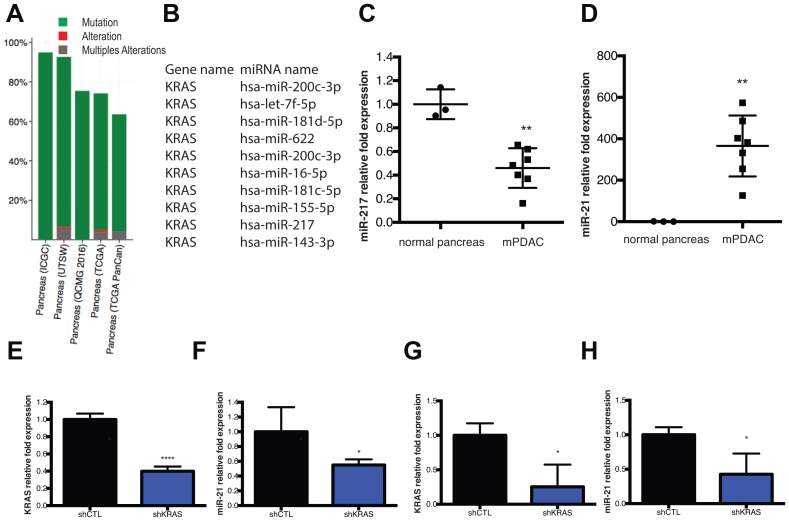
*KRAS* mutation and miRNA dysregulation are components of the mPDAC gene signature. (**A**) cBioPortal data presenting percentage of *KRAS* gene mutation, alteration or multiples alteration map, from 1034 Pancreatic Adenocarcinoma samples from 5 independent studies. (**B**) The top 10 miRNAs that are validated to target the *KRAS* 3′UTR were identified in DIANA-TarBase v8. (**C**) qPCR analysis of miR-217-5p expression level in mPDAC tumor samples compared to normal pancreas. (**D**) qPCR analysis of miR-21-5p expression level in mPDAC tumors samples compared to normal pancreas. (**E**) qPCR analysis of *KRAS* expression levels in D8-175 cells treated with doxocycline for 48 h (shKRAS) compared to untreated D8-175 (shCTL). (**F**) qPCR analysis of miR-21-5p expression levels in D8-175 cells treated with doxycycline for 48h (shKRAS) compared to untreated D8-175 (shCTL). (**G**) qPCR analysis of miR-21-5p expression levels in mPDAC tumors from mice feed with doxycycline. ^*^ *P* = 0.01–0.05; ^**^ *P* = 0.001–0.01; ^***^ *P* < 0.001; ^****^ *P* < 0.0001 N.S., not significant, two-tailed *t*-test.

*KRAS* mutations and alterations represent the most common abnormalities found in human PDAC samples (Figure 1A) [22, 23]. It has also been extensively reported that various miRNAs are dysregulated in PDAC in human and mouse. Among these miRNAs, some are known to target tumor suppressors or oncogenes like *KRAS*. Ten miRNAs have been validated to bind to the *KRAS* 3′UTR in human samples (Tarbase ref), including miR-217 (Figure 1B). Recently, our lab reported on a list of 13 miRNAs significantly deregulated in human PDX PDAC samples [9], and miR-217 was the most downregulated compared to normal samples. In this new study, we used various PDAC models including human and mouse cell lines to test the role of miR-217. In our previous study, we investigated the effect of anti-miR-21 therapy on various models including the D8-175 mouse cell line (mPDAC) derived from *KrasLSL-G12D/+, p53fl/fl* (KC) transgenic mice that bear the most common mutations found in PDAC patients (*KRAS* and *TP53*). We also performed an *in vivo* study in which NOD/SCID mice grew mPDAC allografts after subcutaneous injection of 500,000 mPDAC cells/flank. We measured miRNAs levels in tumors collected from these mice using qPCR by comparing 3 normal pancreases from mice (normal pancreas) to that of 7 mPDAC samples. As compared to our PDX human profiling, miR-217 was downregulated in this PDAC mouse model (Figure 1C) and miR-21 was upregulated (Figure 1D). These results reveal a strong miR-21 and miR-217 PDAC signature in our samples and point to a lack of *KRAS* inhibition by its repressor: miR-217-5p.

Since we wished to test targeting of both *KRAS* and miR-21 for PDAC therapy, we analyzed miR-21 regulation during partial knockdown of *KRAS*. To this end, mPDAC cells carrying an inducible anti-*KRAS* shRNA construct were cultivated under doxycycline selection for 48h. At the end of the treatment, we analyzed *KRAS* expression and miR-21 expression by qPCR. *KRAS* expression was decreased by 60% (Figure 1E) and miR-21 expression was also decreased by 55% (Figure 1F). In the mPDAC model (mPDAC cells carrying an inducible anti-*KRAS* shRNA construct grown as allografts in mice) we also observed miR-21 downregulation after mice were fed with doxycycline food to induce the shRNA *KRAS* (Figure 1G). These results suggest that *KRAS* expression influences miR-21 expression in PDAC and reinforces the idea of testing dual targeting of *KRAS* and miR-21 as a possible therapeutic strategy.

### Reintroduction of miR-217 act as a tumor suppressor targeting KRAS in PDAC

**Figure 2:**
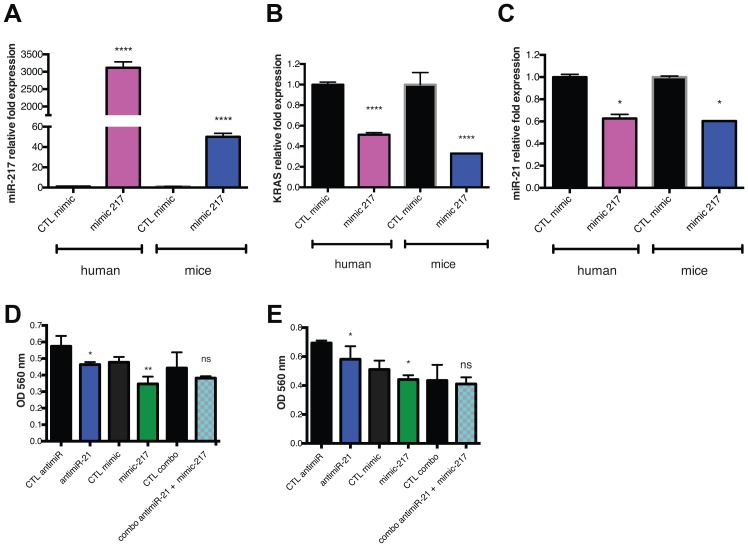
Reintroduction of miR-217 act as a tumor suppressor targeting KRAS in PDAC. (**A**) qPCR analysis of miR-217-5p expression levels in PDAC cell lines (human: PANC1 or mice: D8-175) after 48 h of mimic-217 (or mimic control) transfection. (**B**) qPCR analysis of *KRAS* expression level in PDAC cell lines (human: PANC1 or mice: D8-175) after 48 h of mimic-217 (or mimic control) transfection. (**C**) qPCR analysis of miR-21-5p expression level in PDAC cell lines (human: PANC1 or mice: D8-175) after 48h of mimic-217 (or mimic control) transfection. (**D**) Cell metabolic activity measurements from MTT assays at 24 h after treatment (anti-mir-21 or mimic-217 or combo and associated controls) in D8-175 cells: TPN-NT is TPN forming with scramble RNA - double TPN-NT is 2X concentration of scramble RNA as an appropriate control for toxicity of dual treatment. (**E**) Cell metabolic activity measurements from MTT assays at 24h after treatment (anti-mir-21 or mimic-217 or combo and associated controls) in PANC1 cells. Scale bars, 50 μm Error bars, mean ± s.d ^*^ *P* = 0.01–0.05; ^**^ *P* = 0.001–0.01; ^***^ *P* < 0.001; ^****^ *P* < 0.0001 N.S., not significant, two-tailed *t*-test; *n* = 3 biological replicates.

Because miR-217 was previously validated to target the *KRAS* 3′UTR (Figure 1B) we decided to investigate if the reintroduction of miR-217 could modulate *KRAS* expression in our PDAC models. To this end, we used a chemically modified double-stranded RNA that mimics endogenous miRNA miR-217 (mimic-217). hPDAC (PANC1) and mPDAC (d8-175) cells were transfected with mimic-217 for 48h. At the end of the treatment, expression of miR-217 was analyzed by qPCR. In both cell lines, mimic-217 induced an upregulation of miR-217 from 50 to 3000-fold (D8-175 and PANC1 respectively) (Figure 2A). Concomitantly, *KRAS* expression was monitored by qPCR and both cell lines showed an inhibition of *KRAS* expression level (50-60%) (Figure 2B). These results show that mimic-217 represses *KRAS* in PDAC cells.

Interestingly, we showed that *KRAS* KD promoted miR-21 downregulation in mPDAC cells (Figure 1E and 1F). To verify if the *KRAS* targeting miRNA (mimic-217) promotes the same phenotype, we assessed miR-21 expression level in both cell lines after transfecting mimic-217. It appears that reintroduction of miR-217 promotes miR-21 downregulation (~40%) as observed previously after 48h of shKRAS induction (Figures 2C and 1F). As miR-217 acts as a tumor suppressor and seems to be an interesting target for PDAC therapy we also investigated the effect of its reintroduction (alone or combined with anti-miR-21) on cell viability. After 48h of transfection with either antimiR or mimic, we measured metabolic activity using an MTT assay. Analysis of the results shows a significant inhibition of viability after miR-217 reintroduction in both cell lines. On the other hand, combining anti-miR-21 and mimic-217 did not significantly influence the viability compared to each treatment used alone. These results suggest that dual targeting of these two key miRNAs (21 and 217) may not be the optimal option for PDAC therapy and suggest that direct *KRAS* KD using siRNA (si-KRAS) could be more appropriate than mimic-217 to impair crosstalk in miRNA/mRNA/target regulations.

### Combining antimiR-21 and si-KRAS increases apoptosis and enhances the anti-proliferative effect in PDAC cells and organoids

**Figure 3:**
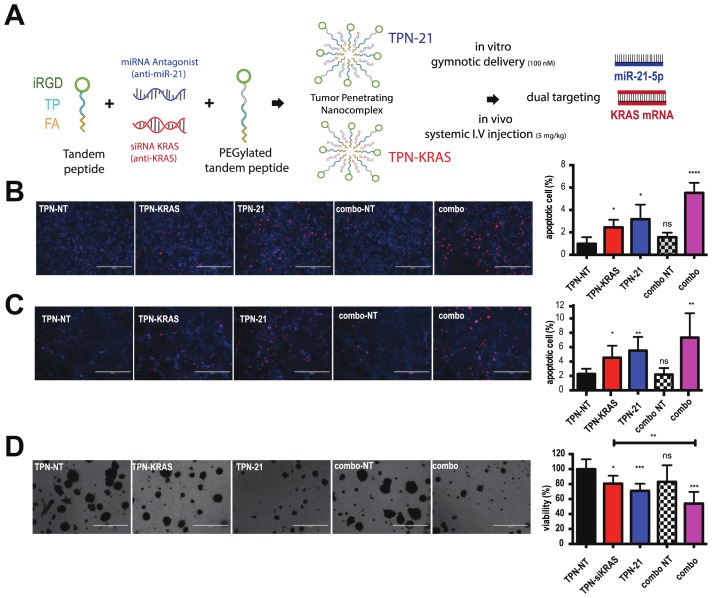
Combining antimiR-21 and si-KRAS increases apoptosis and enhances the anti-proliferative effect in PDAC cells and organoids. (**A**) Representation of tumor-penetrating nanocomplexes (TPN) loaded with anti-miR-21 (TPN-21) or loaded with siRNA-anti-*KRAS* (TPN-KRAS) for dual targeting of miR-21-5p and *KRAS* mRNA. (**B** and **C**) Representative pictures of PANC1 treated 48h with TPN-21, TPN-KRAS or combo, and associated controls. Scale bars 1000μm. Nuclei were stained with DAPI and NucView™ staining (red) report a significant increase in caspase-3 activity after treatments. Quantification of caspase-3 positive cells (Red) on a total cell (DAPI) is presented as apoptotic cell %. (**D**) Representative images of PDO 286 metabolic activity measured from MTT assay after repeated TPN-21, TPN-KRAS or combo and associated controls treatments. Scale bars 400 μm. Associated quantification is presented as viability %. Error bars, mean ± s.d ^*^ *P* = 0.01–0.05; ^**^ *P* = 0.001–0.01; ^***^ *P* < 0.001; ^****^ *P* < 0.0001 N.S., not significant, two-tailed *t*-test; *n* = 3 biological replicates.

As previously demonstrated by our group, in order to optimize delivery and targeting of RNA-therapeutics to PDAC, we have encapsulated antimiRs and siRNA in tumor-penetrating nanocomplexes (TPNs) that have increase tumor targeting through sequential binding to integrins and NRP1 by tumor penetrating peptide iRGD [9, 10]. Here we used TPNs containing anti-miR-21 (TPN-21, blue) as well as an siRNA against *KRAS* (TPN-KRAS, red). Both TPNs were delivered by gymnotic delivery *in vitro* and systemic intravenous repeat injections *in vivo* (Figure 3A). In order to assess the anti-tumor effects of dual anti-miR-21 and si-KRAS therapy in PDAC, we treated cells using either TPN-21, TPN-KRAS, or a combination of both and we compared that to TPN-NT (scramble RNA) or double TPN-NT (combo-NT). After a 48h treatment, we stained for apoptosis by using a specific dye that is cleaved in the nucleus under a high level of activated caspase-3. Analysis of the staining showed a significant increase of caspase-3 activity after either TPN-KRAS or TPN-21 used alone compared to TPN-NT in hPDAC and mPDAC (Figure 3B and 3C). Interestingly, an additional increase of caspase-3 activity is observed with the combination of TPN-21 and TPN-KRAS together compared to that of individual treatments. No significant change was observed between a single and double dose of TPN-NT, indicating the low toxicity of the particles (Figure 3B and 3C).

In order to validate these results for possible human applications, PANC1 cells were plated to promote spheroid formation and relative growth rate was analyzed by performing a metabolic activity measurement using MTT. Analysis of the viability shows similar results to those previously observed in 2D culture including a significant increase of anti-tumor effects after dual targeting of miR-21 and *KRAS* compared to TPN-KRAS alone and combo-NT (Figure 3D). Altogether these promising data suggest that dual targeting of an oncomiR (miR-21) and an oncogene (KRAS) should improve the efficiency of TPN-21 and TPN-KRAS used alone for PDAC *in vivo*.

### Combining TPN-21 and TPN-KRAS shows limited benefit to the tumor growth volume but promotes tumor regression in mPDAC

To test whether *the use of dual miR-21 and KRAS targeting using RNA-based therapy can disrupt* tumor maintenance, we tested our combo-therapy *in vivo*. We generated a PDAC mouse model by injecting 500,000 mPDAC cells/flank in NOD/SCID mice as previously described by our group [9]. As observed from our prior 3D analysis in hPDAC (Figure 3D), repeated, systemic administration of both TPN-21 and TPN-KRAS slowed tumor growth all along the treatment in mPDAC (not shown). In this study, tumor groups were internally heterogenous and some of TPN-NT tumors (as well as in other groups) was very small (smaller <25mm3) when some other are bigger (>169mm3). Tumors sizes are not significantly different at the beginning of the study (Supplementary Figure 1).

**Figure 4:**
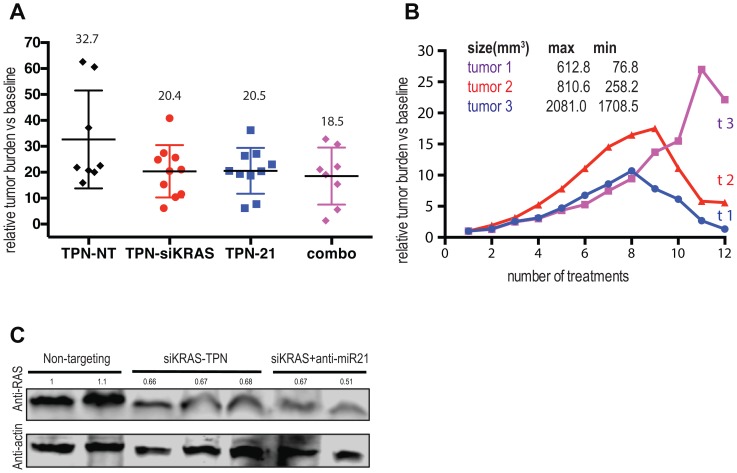
Combining TPN-21 and TPN-KRAS shows limited benefit to the tumor growth volume but promotes tumor regression in mPDAC. (**A**) Relative tumor burden in fold change after 12 I.V injections of TPN-control and combo *n =* 8 or TPN-21 and TPN-KRAS *n* = 10 (5 mg/kg). (**B**) Relative tumor burden in fold change of the 3 tumors showing tumor regression (along 12 I.V injections of combo). Maximum tumor volume measured is indicated (max) as well as the tumor size at the end of the treatment (min). (**C**) Western blot analysis of KRAS expression, Column 1-2 (Non-targeting), columns 3–5 (TPN-siKRAS) columns 6–7 (TPN-siKRAS+TPN-21). Western blot band corresponding to KRAS expression and have been quantified against actin levels. Error bars, mean ± s.d ^*^ *P* = 0.01–0.05; ^**^ *P* = 0.001–0.01; ^***^ *P* < 0.001; ^****^ *P* < 0.0001 N.S., not significant, two-tailed *t*-test; *n* = 3 biological replicates.

Tumor size measurement at the end of the treatments revealed that delivery of anti-miR-21, as well as si-KRAS, significantly reduced mPDAC tumor growth by 37.3% and 37.6% respectively (Figure 4A). In addition, after dual targeting of miR-21 and KRAS (combo), the final tumor growth was reduced by 43.4%, revealing an additional 6% anti-tumor effect (Figure 4A). Evaluation of the tumor growth volumes of each mouse throughout the treatment highlighted some dramatic changes in tumor growth behavior in the group receiving the combination of anti-miR-21 and siRNA anti-*KRAS*. As shown (Figure 4B), only 3 tumors displayed size regression (from 17.93% to 87.59% (tumors 3 and 1 respectively)) and all 3 were in the dual targeting treatment group (Supplementary Figure 2). KRAS expression analysis of tumor samples after TPN treatments (non-targeting, TPN-siKRAS and dual treatment TPN-siKRAS+TPN-21) was performed by Western blot and we found. that the dual treatment (TPN-siKRAS + TPN-21) did not enhance KRAS KD compared to that of regular siKRAS KD (TPN-siKRAS) (Figure 4C).

This result paves the way to continue exploring how dual targeting of miR-21 and KRAS can promote tumor regression in mice and generates additional questions regarding the specific oncogene and oncomiR profile of the 3 tumors that demonstrated tumor regression.

## DISCUSSION

In this study, we tested the dual targeting of an oncomiR (miR-21-5p) and an oncogene (*KRAS*) for PDAC therapy. This unique and innovative combination of compounds builds on trends in current research of successful combination approaches to PDAC therapy. To date, use of a classical chemotherapeutic agent like gemcitabine with another compound (erlotinib, nab-paclitaxel, specific pseudopeptide or small non-coding RNA strategy) has remained the first therapeutic research option for PDAC [3–6, 9]. As PDAC specimens (mouse and human samples) present with a strong *KRAS* mutation profile associated with a strong miRNA dysregulation profile (miR-21-5p and miR-217-5p), we decided to test dual targeting of miR-21-5p and a natural *KRAS* targeting miRNA (miR-217-5p) (Figures 1 and 2) [11, 15].

Even though reintroduction of miR-217-5p using a chemically modified double-stranded RNA that mimics endogenous miRNA miR-217 (mimic-217) in mPDAC and hPDAC was successful and promoted significant *KRAS* knockdown and a viability defect, dual utilization of miR-217-5p and anti-miR-21-5p did not yield stronger results than the individual treatments (Figure 2D, 2E). These observations could be in part due to the higher toxicity of mimic control than antimiR control that decreases the viability of the double control in our assay, resulting in a minimization of the effect (Figure 2D, 2E). On the other hand, it is not surprising that indirectly targeting *KRAS* using a miRNA promoted weaker knockdown than direct targeting with an siRNA (Figure 3B, 3C). For the rest of our combination study of oncomiR and oncogene targeting, we used an siRNA to target *KRAS* instead of mimic-217.

Simultaneous targeting of these two notorious PDAC drivers was achieved by using the PEGylated iRGD TPNs nanoparticle-based approach to deliver both nucleic acids (anti-miR-21 and siRNA anti-*KRAS*) in 2D, 3D and *in vivo* models. This compound was previously described by our groups to surmount the PDAC barrier and promote safe delivery of nucleic acid compounds [9, 10]. Our study highlights a benefit of dual targeting of these two PDAC drivers compared to single treatment and very interestingly, we even recorded 3 tumors regressing in dual-treated mice. These results indicate that dual targeting of *KRAS* and miR-21-5p could potentially abolish tumor growth in PDAC. Nevertheless, even though we observed a general slowing of tumor growth in the 8 mice from the combination group (combo), only 3 of them display a dramatic change in the tumor growth behavior. It’s therefore unclear if the combined treatment variability is due to overall inconsistent TPN delivery to the tumor or by variability in the functional activity of the siRNAs and antimirs themselves and will be investigated in the futur. So far, we can potentially hypothesize that like humans, each mouse presents its own gene profile including specific oncogene and oncomiR dysregulation patterns and that dual targeting of miR-21-5p and *KRAS* is not the optimal therapeutic option for all of them. In fact, personalized medicine for PDAC and cancer, in general, is currently a major challenge. As also mentioned in our previous study, there is a strong disconnect between pre-clinical work performed in cell lines and xenografts and success of clinical trials due to the heterogeneity of patient gene expression pattern including oncogene and oncomiR expression [24]. For this reason, to better understand why these 3 tumor samples show regression after dual treatment we would have had to have profiled them before the therapy and then be able to understand and eventually predict tumor response. None-the-less, this specific phenotype suggests that additional work will be needed to optimize this strategy in the future.

## MATERIALS AND METHODS

### Tumor mouse models

For the generation of mPDAC mouse models, bilateral flank allografts were implanted on 6-week old NCR/nude mice each seeded with 5 × 10^5^ cells. Tumor growth was monitored twice a week by observation and palpation. We defined ~100 mm^3^ tumors as the starting point to perform treatment trials. Mice were treated 2 times a week for a total of 8 intravenous injections with either TPN-21, TPN-KRAS, TPN-control (5 mg/kg oligonucleotides) or a combination (TPN-21 and TPN-KRAS). At the end of the treatment, mice were sacrificed, tumors were collected, and samples were processed appropriately. All procedures were conducted following an institutionally approved animal IACUC protocol and as described previously [9, 10].

### qPCR

3D-models and organoids were separated from matrigel by washing with PBS. *Cell lines* and mPDAC tumors were lysed using Trizol and RNA extraction was conducted using the mirVana miRNA Isolation Kit. cDNA synthesis was performed using miScript II RT Kit (Qiagen) with 250 ng input RNA. qRT-PCR was performed using the Roche480 Light Cycler^®^ miRNA system as per manufacturer’s instructions, and gene expression was normalized to U6 and/or 18s. For *KRAS* mRNA analysis, cDNA synthesis was performed using Verso cDNA Synthesis Kit (Life technology) using 500–1000 ng input RNA. cDNA was used for SYBR Green-based real-time PCR. Gene expression was normalized to GAPDH. Hsa-miR-21-5p, mmu-miR-21-5p, Hsa-miR-217-5p, mmu-miR-217-5p, U6 and 18s miScript Primers were obtained from Qiagen.

The *KRAS* primers used for mouse and human are F:AGAGGACTCCTACAGGAAACAAGTAGTAATTGAT

R:AGCCCTCCCCAGTTCTCATGT

### Cell lines

Human PDAC cells PANC-1, were obtained from the American Type Culture Collection (ATCC), and cultured at 37°C with 5% CO_2_ in DMEM, supplemented with 10% FBS and 1% penicillin/streptomycin. Mouse PDAC cell line D8-175 is derived from *Pdx1-Cre; Kras^+/LSL-G12D^; Trp53 ^fl/fl^* (KPC) mice, and was cultured in DMEM supplemented with 10% FBS and 1% penicillin/streptomycin. For *in vivo* tumor development tracking, D8-175 cells were *luc+* (luciferized) and carried a doxycycline inducible shRNA against *KRAS*. Cell lines were tested for mycoplasma using the Lonza MycoAlert™ Detection Kit.

### AntimiR, mimic and siRNA transfection

For RNA-based functional experiments, mirVana^®^ miRNA inhibitor for hsa-miR-21-5p and mmu-miR-21-5p and *mir*Vana^®^ miRNA mimic for hsa-miR-217-5p and mmu-miR-217-5p as well as controls mirVana™miRNA Inhibitor and *mir*Vana™ miRNA Mimic, Negative Control #1 (Life Technologies) were used at 50 nM and transfected into cells using RNAiMAX (invitrogen), as per manufacturer’s protocol. *KRAS* siRNAs were synthesized by Dharmacon (GE Healthcare) with ON-TARGETplus specificity enhancement. The following sequences were used (given as the sense strand without overhangs): siKras.476 against murine and human *KRAS*: 5′-ACCAUUAUAGAGAACAAAUUA-3′, siNC non-targeted control: 5′-UUCUCCGAACGUGUCACG UUU-3′.

### Organoids

Tumor organoids were generated as described by Huang et al 2015 [19]. Fresh PDX tumor fragments were minced with a No.22 blade into 1-2 mm small pieces then digested with 1mg/ml collagenase/dispase for 30 minutes, followed by Accutase digestion for 40 minutes. The slurry was re-suspended in DMEM then filtered through a tissue strainer and centrifuged at 1500 rpm for 5 minutes. After aspirating the supernatant, cell pellets were re-suspended in organoid growth medium supplemented with 5% Matrigel and 10 uM Y27632. Organoids were grown under standard culture conditions (5% CO2, at 37 °C). Organoids were treated with TPN-21, TPN-KRAS, TPN-control or combo (100 nM) following a QOD treatment plan.

### Viability and apoptotic assay

Viability assay on cells and organoids was performed by using MTT reagents from SIGMA (M2128) according to the manufacturer’s recommendation. The apoptosis assay was performed by using NucView™ 530 Caspase-3 Substrate (Biotium). 3D-models were briefly incubated with NucView reagent (1/500) and Hoescht (1/1000) for 30-60 minutes and the red signal corresponding to caspase-3 positive structures were counted and normalized to the total number of structures. Viability and apoptosis of 3D-models and organoids after treatment was conducted on > 3 independent wells per condition in each experiment for > 3 independent experiments.

### Tumor penetrating nanocomplex and binding assay

Tandem peptide (pTP-iRGD: CH3(CH)15-GWTLNSAGYLLGKINLKALAALAKKIL-GGK (TAMRA)GGCRGDKGPDC, Cys-Cys bridge) was synthesized by CPC Scientific. PEGylation method of the tandem peptide was previously described [20]. All siRNAs were synthesized by Dharmacon (GE Healthcare) with ON-TARGETplus specificity enhancement. The sequences used were as follows (sense strand, without overhangs): siKras: 5′-GGAAACCUUCUUUUUUCUAAG-3′, siNC non-targeted control: 5′-UUCUCCGAACGUGUCA CGUUU-3′. mirVana™ miRNA inhibitor against hsa-miR-21-5p and non-targeting controls were obtained from Thermo Scientific. To assembly the TPNs in solution, oligonucleotides, PEGylated pTP-iRGD, and pTP-iRGD were resuspended in nuclease-free water and mixed with the oligonucleotides, PEG-containing component, and peptide in 1:2.5:15 molar ratios. This was achieved by first thoroughly mixing the oligonucleotides with the PEGylated pTP-iRGD and subsequently mixing in the peptide to create a concentrated solution of TPNs that were adjusted to the desired dilution and buffer composition with appropriate diluent. Binding of iRGD-TAMRA on cells was performed at 4°C on ice.

### Western blotting

Subcutaneous tumors driven by D8-175 cell line were isolated, cryogrinded and lysed in 1x RIPA buffer with protease and phosphatase inhibitor (Thermo Scientific) for 30 minutes on ice. The whole-cell lysates were then clarified by centrifugation for 25 minutes at 15,000 rpm at 4° C. Protein concentrations were determined using the BCA Protein Assay Kit (Pierce). Equal amounts (30 μg) of protein samples were fractionated by a Novex^®^ 4–20% Tris-Glycine gel (Thermo Scientific), transferred to nitrocellulose membranes (Thermo Scientific), and blocked in Odyssey blocking buffer (LI-COR Biosciences). KRAS was probed by primary rabbit antibody (1:1000) (Cell Signaling, #3965), and actin was stained using primary rabbit antibody (1:1000) (Abcam, ab8227). The desired bands were detected by labeling with anti-rabbit (1:10,000) IgG-IRDye 680 secondary antibodies and visualized using the Odyssey Infrared Imaging System (LI-COR Biosciences).

### Statistical analysis

Statistical analysis was achieved by using a two-tailed unpaired t test on GraphPad Prism software. 2-way ANOVA were completed for miRNA profiling assays. All graphs show means ± S.D ^*^ *P* = 0.01–0.05; ^**^ *P* = 0.001–0.01; ^***^ *P* < 0.001; ^****^ *P* < 0.0001 N.S., not significant. For qRT–PCR data, means and s.d. were calculated at the ΔΔCt level before being converted to fold changes as presented in the graphs [21].

## SUPPLEMENTARY MATERIALS


