# Supplementary material for: Tumor penetrating nanomedicine targeting both an oncomiR and an oncogene in pancreatic cancer
Source: Oncotarget. 2019 Sep 3;10(51):5349–58. doi: 10.18632/oncotarget.27160 (PMC6731108; doi:10.18632/oncotarget.27160)
Supplement: Supplementary file 1 [file oncotarget-10-5349-s001.pdf]

Tumor penetrating nanomedicine targeting both an oncomiR and an oncogene in pancreatic cancer

SUPPLEMENTARY MATERIALS

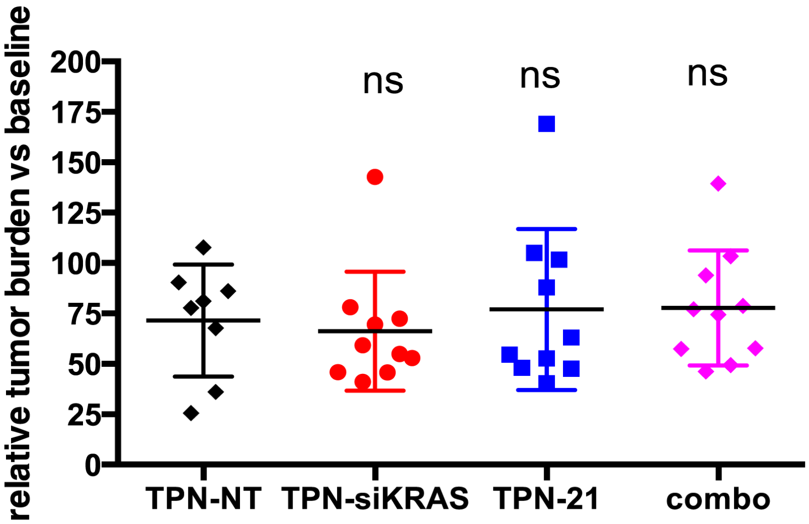

Supplementary Figure 1: Tumors sizes are not significantly different at the beginning of the study.

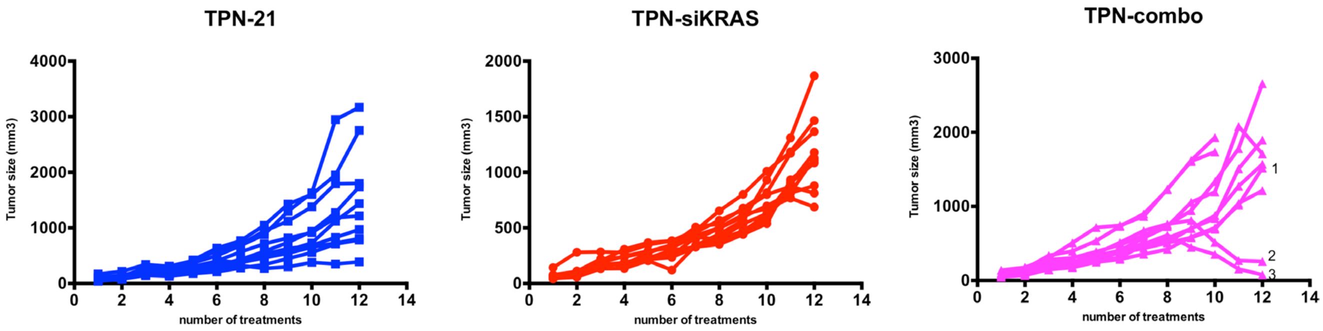

Supplementary Figure 2: Time course for all tumors in each treatment class.
